# Beyond the Excluded Volume Effects: Mechanistic Complexity of the Crowded Milieu

**DOI:** 10.3390/molecules20011377

**Published:** 2015-01-14

**Authors:** Irina M. Kuznetsova, Boris Y. Zaslavsky, Leonid Breydo, Konstantin K. Turoverov, Vladimir N. Uversky

**Affiliations:** 1 Laboratory of Structural Dynamics, Stability and Folding of Proteins, Institute of Cytology, Russian Academy of Sciences, 4 Tikhoretsky Ave., St. Petersburg 194064, Russia; E-Mails: imk@mail.cytspb.rssi.ru (I.M.K.); kkt@incras.ru (K.K.T.); 2 St. Petersburg State Polytechnical University, 29 Polytechnicheskaya st., St. Petersburg 195251, Russia; 3 Cleveland Diagnostics, 3615 Superior Ave., Suite 4407B, Cleveland, OH 44114, USA; E-Mail: Boris.Zaslavsky@Cleveland-Diagnostics.com; 4 Department of Molecular Medicine and USF Health Byrd Alzheimer’s Research Institute, Morsani College of Medicine, University of South Florida, 12901 Bruce B. Downs Blvd. MDC07, Tampa, FL 33612, USA; E-Mails: lbreydo@health.usf.edu; 5 Biology Department, Faculty of Science, King Abdulaziz University, P.O. Box 80203, Jeddah 21589, Saudi Arabia

**Keywords:** macromolecular crowding, excluded volume effect, viscosity, soft interactions, solvent properties, aqueous two-phase system, partition

## Abstract

Macromolecular crowding is known to affect protein folding, binding of small molecules, interaction with nucleic acids, enzymatic activity, protein-protein interactions, and protein aggregation. Although for a long time it was believed that the major mechanism of the action of crowded environments on structure, folding, thermodynamics, and function of a protein can be described in terms of the excluded volume effects, it is getting clear now that other factors originating from the presence of high concentrations of “inert” macromolecules in crowded solution should definitely be taken into account to draw a more complete picture of a protein in a crowded milieu. This review shows that in addition to the excluded volume effects important players of the crowded environments are viscosity, perturbed diffusion, direct physical interactions between the crowding agents and proteins, soft interactions, and, most importantly, the effects of crowders on solvent properties.

## 1. What Is the Macromolecular Crowding and How Can It Be Modeled?

Cells are tightly packed with various biological macromolecules (proteins, protein complexes, nucleic acids, ribonucleoproteins, polysaccharides, *etc.*), suggesting that the intracellular environment is extremely crowded and has rather limited free space. In fact, estimates showed that the total intracellular concentration of biological macromolecules ranges from 80 to 400 mg/mL [1,2,3], that these macromolecules occupy 5%–40% of cellular volume [4], and that all this creates a unique crowded medium with considerably restricted amounts of free water [1,5,6,7,8,9]. Although intracellular media contain billions of protein molecules [10] and very large quantities of DNA, RNA, ribonucleoprotein, and polysaccharide molecules, typically, no individual macromolecular species is present there at very high concentrations. As a result, intracellular environment is typically described as “crowded” rather than “concentrated” [7,9,11]. Such a crowded medium resembles very thick molecular soup, being characterized by high volume occupancy, where the average distances between macromolecules could be noticeably smaller than the size of the macromolecules themselves [12]. Since two macromolecules cannot be at the same time in the same place, the excluded volume (*i.e.*, the volume occupied by macromolecules and unavailable to other solutes) is generated. In fact, this excluded volume originates from the mutual impenetrability of solute molecules that generates steric repulsion. Obviously, such crowed environments might significantly affect any reaction the course of which is dependent on the available volume [5,13]. Furthermore, such crowded environment is expected to place constraints on the active factors present in the microenvironment [8,14,15]. The thermodynamic consequences of the unavailable volume are described in terms of excluded volume effects [5,14].

Based on these considerations, it is obvious that the relatively ideal thermodynamic conditions typically used for the *in vitro* analysis of protein structure and function (where a protein of interest is dissolved at a low concentration in warm slightly salted water) cannot be used even as a first approximation of the “real life” intracellular protein environment with its highly packed milieu. However, there is an increasing appreciation of this shortcoming, and the view that the macromolecular crowding is an important phenomenon that should be taken into account in biochemical studies is gaining attention [5,8]. It is accepted now that the macromolecular crowding effects on biological macromolecules of interest may be examined experimentally by using concentrated solutions of some model “crowding agents”, such as poly(ethylene glycol), Dextran, Ficoll, or inert proteins [14,16]. Although concentrated solutions of such polymers can mimic macromolecular crowding characterized by high excluded volume, it is important to remember that the efficiency of the artificial macromolecular crowding is expected to depend on a ratio between the hydrodynamic dimensions (or occupied volumes) of the crowder and the test molecule, with the most effective conditions being those where the volumes occupied by a crowding agent and a test molecule are comparable [15,17,18].

Another important side of the intracellular crowded environment is molecular confinement. There are at least two experimental approaches to model limited available space of such molecular imprisonment, the encapsulation of a studied protein in silica glass using the sol-gel techniques and encapsulation of a test protein into the reverse micelles. The sol-gel encapsulation is considered as a reliable model of the molecular confinement despite the fact that the fraction of the total volume excluded by the silica matrix is smaller than the fractional volume occupied by macromolecules in living cell [19,20]. However, since the protein itself may dictate the pore size during the gelation process, the sizes of protein-occupied pores in these gels have the same order of magnitude as the diameter of a protein [21]. Although proteins are not bound covalently to the highly porous silica matrix, and although encapsulated macromolecules are unable to escape the glass under most solvent conditions [19], the sol-gel glass matrix does substantially limit the rotational freedom of the protein [22] not limiting the exchange of the solvent that freely permeates the silica matrix. Another important advantage of this approach is the optical transparency of the resulting glass products that allows application of the majority of spectroscopic techniques used to monitor the structure of proteins in dilute solutions, such as fluorescence [23,24] and circular dichroism (CD) [19,20]. Using this approach, the solvent effects on the secondary structure of several ordered proteins have been analyzed by CD following encapsulation in the hydrated pores of a silica glass matrix by the sol-gel method [19,20]. For several globular proteins it has been shown that the conditions resulting from the sol-gel glass encapsulation do not dramatically affect their structure and function [23,25,26]. 

Another approach to study the effect of molecular confinement on the dynamics of both a target protein and solvent molecules is the encapsulation of the protein of interest within the shell of a reverse micelle and dispersing the resulting particle in a low viscosity fluid, such as the short chain alkanes [27,28,29,30,31]. Reverse micelles are unique because they represent nano-size pockets of encapsulated water separated from an organic medium by a surfactant [32,33,34]. The size of the micelles and the resulting degree of protein/peptide hydration inside the reverse micelle can be tuned by varying the ratio of water to the concentration of the surfactant (e.g., sodium bis(2-ethylhexyl) sulfosuccinate, AOT) in the organic solvent [31,35]. Adding water increases the size of the micelle, permitting an examination of the effect of a systematic enhancement of the number of hydration waters surrounding a confined protein [32,36]. Similar to the sol-gel glass encapsulation, structural properties of proteins in reverse micelles can be analyzed by various spectroscopic techniques, such as fluorescence, CD [33], and NMR [27,28,29,30,31].

Protein and peptides can also be encapsulated in mesoporous silica with tunable pore diameter and easily functionalized surface [37,38], in silica tubes [39] or carbon nanotubes [40], inside the cavities of polyacrylamide gels [41,42,43,44] or weakly hydrophobic pores of methacrylate resin [45], or in self-assembled organic nanotubes [46].

Although all aforementioned encapsulation techniques provide suitable conditions for studying the effects of molecular confinement on different aspect of protein structure and dynamics and on the solvent dynamics and properties, the use of solutions with high concentrations of inert polymers represents the most common approach to model the various effects of macromolecular crowding.

## 2. What Can Crowding Do to a Protein?

Even in the absence of direct interactions between macromolecules, the ability of macromolecules to occupy space, and thereby to exclude other molecules from their neighborhoods, can have a profound effect on tertiary structure, quaternary structure, and the kinetics of enzyme-catalyzed reactions [47]. Using various *in vitro* systems with model crowded conditions, it has been established that macromolecular crowding might have large effects on conformational stability and structural properties of biological macromolecules [9,11,19,20,48]. Also, macromolecular crowding may affect various physico-chemical equilibria, such as protein folding [47,49], binding of small molecules [50], enzymatic activity [50], protein-protein interactions [11], protein compaction [51], as well as pathological protein aggregation and extent of amyloid formation [16,52,53]. In a recent review, we considered these and other consequences of macromolecular crowding [50]. It has been emphasized that it is almost impossible to find a single protein-related feature that would not be affected by the addition of high concentrations of macromolecules. In fact, biological activities, structural features of ordered and intrinsically disordered proteins, shapes of protein molecules, protein dynamics, protein self-assembly, protein assembly and ability to interact with various partners or to form functional or pathogenic complexes, protein oligomerization, protein aggregation, protein fibrillation, protein folding, structural stability of proteins, their conformational behavior, phase separation and compartmentalization, as well as many other protein-related aspects can be changed in the crowded milieu [50]. 

## 3. Excluded Volume Effects

Section below represents an oversimplified representation of the molecular crowding effect. The readers interested in the formal and precise description of this phenomenon are recommended to look into the original papers and reviews [5,17,54,55,56]. Furthermore, a number of specialized reviews describing various effects of macromolecular crowding on biological macromolecules were published during the past decade [47,57,58,59,60], and therefore there is no need to recapitulate published material. 

### 3.1. Excluded Volume Simplified

Excluded volume phenomenon originates from a very simple consideration that two molecules cannot occupy the same space in solution. As a result of steric hindrance or impediment, each macromolecule is expected to exclude other molecules from its neighborhood [47]. In a simplest case of spherical particles, positions of which in the space are completely specified by the position of their centers, the closest two such molecules can approach each other is a distance equal to the sum of their radii. This generates a spherical excluded volume around each molecule which is inaccessible to the centers of all the other molecules [47]. In solutions with increasing concentrations of such spherical particles, the number of ways that can be used to place added molecules is progressively limited. In other words, the volume of solution available to the molecules is restricted to the part of space from which they are not excluded [47]. This obviously decreases the randomness of the distribution of such particles in concentrated solutions. Therefore, from the viewpoint of thermodynamics, the entropy of the crowded solution is noticeably decreased in comparison with the entropy of ideal solution where solute molecules occupy no space. As a consequence of this entropy decrease, the free energy of the solute increases [47]. This increase in free energy arising from volume exclusion can be viewed as a reflection of the increase in the thermodynamic activity of solute, and therefore is expected to affect various processes determined by the activity [47]. In agreement with this model, it was shown that when the concentration of hemoglobin increases to the levels observed *in vivo* in erythrocyte (~320 g/L), its osmotic pressure rises dramatically and the thermodynamic activity of hemoglobin reaches ~100 times its concentration [61].

Notably, it was hypothesized that excluded volume effects do not only influence the thermodynamic activity of the concentrated solute itself but also might affect other solutes present in the solution at low concentrations [47]. In other words, behavior of a molecule of interest in the non-ideal solution containing high concentrations of any inert “bystander” molecules is expected to be different from the behavior of this molecule in the ideal solution since the “bystander” molecules will contribute significantly to the overall degree of volume occupancy in the solution and thereby to the non-ideality of solution [47]. Among potential targets of these excluded volume effects are chemical equilibria (such as protein folding-unfolding reaction), association reactions, and enzymatic kinetics. The formal quantitative description of these effects is given in [47]. Since the detailed analysis of the literature on the behavior of various proteins in crowded milieu is given in our recent review [50], below we provide just a few examples to illustrate the corresponding points. 

### 3.2. Effect of Excluded Volume on Chemical Equilibria 

#### 3.2.1. Protein Folding and Conformational Stability

The application of the excluded volume model to a protein folding process (*i.e.*, a transition from a less compact unfolded form to a more compact folded state) suggests that the presence of a space-filling substance might significantly affect the protein folding process by favoring a more compact (folded) state over the unfolded form, and these folding-promoting effects are expected to increase exponentially with the increase in the concentration of the crowding agent [47,49]. In agreement with these predictions, numerous studies clearly showed that macromolecular crowing can affect protein folding process, influence protein conformational equilibrium and thereby modulate the conformational behavior and stability of a protein [50]. 

For example, the kinetic study of the unfolding-refolding reaction of a redesigned apocytochrome *b*_562_ revealed that the folding rate of the protein in mild crowded environments (in the presence of 85 mg/mL of PEG-20000) was significantly accelerated, whereas the unfolded rate remained mostly unchanged [62].

The thermal stability of creatine kinase (CK) was shown to be noticeably enhanced in the presence of high concentrations Ficoll-70 [63]. Furthermore, the increase in the concentration of this crowding agent resulted in an increase in the transition temperature *T_m_* with constant enthalpy change *ΔH_u_* of the thermal denaturation process [63]. Also, the presence of high concentrations of this crowding agent increased the compaction degree of both native and temperature-denatured states of CK, with the denatured ensemble undergoing more noticeable compaction [63]. It was also shown that although recombinant human brain-type creatine kinase (rHBCK) can be efficiently inactivated by 0.5 M guanidinium hydrochloride (GdmHCl) at 25 °C in dilute solutions, the inactivation process was dramatically slowed down by high concentrations of PEG-2000 or Dextran-70 [64]. Here, in the presence of 200 mg/mL PEG-2000, ~35.33% of rHBCK activity was retained after 4 h of incubation at 0.5 M GdmHCl and 25 °C, whereas no rHBCK activity was observed when protein was incubated in the absence of macromolecular crowding agents [64]. 

In another study, reduced and carboxyamidated RNase T1 (TCAM) was shown to be substantially unfolded and not catalytically active in aqueous media [65]. However, the addition of macromolecular crowding agents (400 mg/mL Dextran-70) resulted in the appearance of the catalytically active protein. Since the activity of this crowding-induced form accounted for approximately 16% of the total available TCAM in solution, the authors concluded that macromolecular crowding agents are only modestly effective in promoting folding of this protein [65]. 

Finally, secondary structure and conformational stability of the 148-residue single-domain α/β protein, *Desulfovibrio desulfuricans* apoflavodoxin were shown to be enhanced by Ficoll-70 [66]. For example, high concentrations of this crowding agent dramatically increased the thermal stability of apoflavodoxin (Δ*T*_m_ of 20 °C at 400 mg/mL; pH 7) [66]. Also, the presence of macromolecular crowding agents was shown to lead to the increased amount of secondary structure in the folded states of two structurally-different proteins, α-helical VlsE and α/β flavodoxin [67]. The authors showed that the structural content of flavodoxin and VlsE was enhanced by 33% and 70%, respectively, in 400 mg/mL Ficoll-70 (pH 7, 20 °C) [67]. This crowding-induced “over-folding” was also accompanied by the noticeable increase in the conformational stability of these proteins [67].

#### 3.2.2. Changes in Protein Compaction and Shape

Obviously, high concentrations of inert bystander molecules might affect any chemical equilibrium involving changes in the accessible volume and not only the protein folding reaction. Therefore, among the potential targets that might “feel” the presence of crowding agents are multidomain proteins existing in close and open functional states, or proteins undergoing transitions between elongated and more compact forms. In agreement with these considerations, the addition of Ficoll-70 induced noticeable compaction of phosphoglycerate kinase (PGK, a 415-residue protein containing two nearly equal-sized domains connected by a flexible linker) [68]. This compaction was explained as a transition from an open extended form to a compact closed form of a protein [68]. Analysis of another asymmetric protein, calmodulin (CaM), which is a dumbbell-shaped molecule containing two globular domains (N- and C-lobes) separated by a long linker region, revealed that it is converted into more compact structures in the presence of crowding agents [69]. 

It is expected that macromolecular crowding might affect protein folding by influencing the hydrodynamic volume of the unfolded state. In other words, macromolecular crowding might provide an indirect stabilizing effect to the folded states of proteins due to destabilization of the more extended and malleable denatured states [70,71]. The existence of direct effect of macromolecular crowding on the unfolded state of a protein, forcing it to become more compact at crowded conditions, has been demonstrated for the urea-unfolded small ribosomal protein S16 [72]. This analysis revealed that in the presence of Dextran, the unfolded ensemble of S16 was more compact than in the dilute solutions, and this partially collapsed unfolded form possessed noticeable residual structure [72].

### 3.3. Effect of Excluded Volume on Association Reactions 

In the case of associating molecules, it is also expected that the association equilibrium will be affected by the presence of significant concentrations of the space-filling bystander molecules [47]. Again, in the artificially crowded environment, both the associating macromolecules of interest and the inert bystander molecules exclude each other from their neighborhoods. Since the excluded volume around each dimer of the macromolecule of interest is smaller than twice the excluded volume of each monomer, and since to keep the overall entropy as high as possible the system tends to minimize the total excluded volume (or, alternatively, restore some of the available solution volume lost through the addition of the bystander), the formation of dimers (or high order oligomers and aggregates) will be preferable in the crowded milieu. In other words, the complex system that includes solvent, high concentration of bystander molecules, and some amount of associating molecules of interest will follow the Le Chatelier’s principle stating that when a system originally at equilibrium is perturbed, it will adjust in a direction opposite to the perturbation [47]. In other words, in response to the addition of the bystander molecules, the system will change to minimize the overall crowding by enhancing the association of molecules of interest, thereby reducing the excluded volume [47]. 

Literature contains numerous examples supporting this hypothesis, and many types of association reaction including both functional and pathologic self-oligomerization and aggregation of many proteins were indeed dramatically enhanced in the crowded environments [50]. Furthermore, a noticeable influence of macromolecular crowding on folding-aggregation behavior of many proteins was pointed out. Since long-lived partially folded intermediates accumulating in the unfolding-refolding reaction of many proteins are known to be extremely aggregation-prone, these proteins seem to experience a double hit by the excluded volume. First, the addition of the high concentrations of crowding agents accelerates partial folding of these proteins from extended unfolded state to a more compact folding intermediate with high association potential. Then, to minimize the total excluded volume of a system, these partially folded species self-assemble to form oligomers or other types of aggregates. Formation of such oligomers/aggregates halts protein folding reaction, illustrating that macromolecular crowding enhances protein aggregation at the expense of correct folding [9]. For example, refolding of reduced hen lysozyme was not affected by crowding, whereas the refolding process of the unfolded and reduced protein was almost completely abolished due to the protein aggregation at high concentrations of crowding agents [2]. Similarly, refolding of glucose-6-phosphate dehydrogenase (G6PDH) and protein disulfide isomerase was shown to be hampered by macromolecular crowding due to the formation of soluble aggregates [73]. 

### 3.4. Enzymatic Reactions in Crowded Media 

Since the efficiency of the macromolecular crowding depends on a ratio between the hydrodynamic dimensions of the inert crowder and the test molecule, and since the most effective conditions are achieved when these volumes are comparable [15,17,18], one would expect that the interaction of a substrate with enzyme would not be affected much by the excluded volume since the volume of a substrate is too small. However, if catalysis is accompanied by the changes in the size, or shape, or oligomeric state of an enzyme or when enzyme interacts with a large substrate, e.g., a nucleic acid or a protein, then macromolecular crowding can affect an enzymatic reaction as well [47]. 

In agreement with these predictions, the catalytic efficiency of many DNA- and RNA-modifying enzymes (e.g., DNA ligases from rat liver nuclei, *Escherichia coli* [74], T4 bacteriophage [74], or T4 RNA ligase [75]) was shown to be noticeably increased in the presence of high concentrations of various crowding agents. Also, crowded media were shown to affect the reversed proteolysis and facilitate proteosynthesis of a polypeptide product from non-covalent protein complexes obtained by limited proteolysis of a target protein (e.g., the reformation of native triose-phosphate isomerase (TPI or TIM) from multiple fragments catalyzed by subtilisin) [76]. 

Analysis of the effect of macromolecular crowding on structure and function of a two-domain DEAD-Box helicase eIF4A showed that a crowding-driven shift of the conformational equilibrium of eIF4A from an inactive open state towards the closed active conformation was responsible for the enhancement of the ATPase activity of this translation initiation factor [77]. 

Very compelling evidence is accumulated on the effect of excluded volume on catalytic activity of oligomeric enzymes [50]. For example, in the absence of crowding agents, rabbit muscle glyceraldehyde-3-phosphate dehydrogenase (GAPD) exists as a mixture of very active monomers and much less active tetramers. Crowded environment shifted the monomer-tetramer equilibrium dramatically favoring the formation of less active tetramers [54]. Crowding resulted in a dramatic stabilization and enhanced catalytic activity of a multienzyme complex, bacterial phosphoenolpyruvate carbohydrate phosphotransferase system (PTS) [78,79]. 

## 4. Crowded Environments beyond the Excluded Volume Effects

Although many experimental and computational studies suggested that the excluded volume model provides reasonable phenomenological description of the effects of crowded media on various aspects of protein behavior, the evidence is accumulating in favor of the important conclusion that some other aspects of such complex solutions should be taken into account, too. For example, based on the calorimetric analysis of the ubiquitin thermal unfolding in the presence of different cosolutes, such as glucose, KCl, urea, Dextran, and PEG, it has been concluded that contrarily to the excluded volume theory stating that the behavior of a protein in crowded environments is determined by the hardcore steric repulsions among the macromolecules and therefore should have a clear entropic origin, the existence of an enthalpic stabilization and an entropic destabilization for glucose, Dextran, and PEG has been established [80]. Curiously, Dextran and PEG had different effects on the ubiquitin stability in these experiments, with Dextran showing enthalpic stabilization and with entropy dominating over enthalpy thereby leading to an overall destabilization in the presence of PEG [80]. Obviously, there are several aspects beyond the excluded volume effects that constitute a basis for the mechanistic complexity of crowded environments, and some of these aspects are considered below.

### 4.1. Not All Crowding Agents Are Created Equal 

In the excluded volume model, the chemical nature of the bystander molecules does not play a role, since phenomenon is described solely based on the volume occupied by these molecules. From this viewpoint, inert molecules of different chemical nature but with similar hydrodynamic volumes should affect behavior of a protein of interest in a similar way. However, several studies showed that not all crowding agents are created equal and some solutes are more effective crowders than other solutes [50]. Elcock pointed out that there is an important question of whether truly inert crowding agents that could be used in experiments to provide a direct read out of excluded volume effects only actually exist, or whether it is inevitable that all crowding agents will also cause additional effects that must be considered [81]. It was rightly pointed out that the presence of surface charges, site-specific interactions, and an intricate surface topology could make proteins a more complex and biologically relevant crowding agent than any simple polymer [82]. For example, comparative study of the enzymatic activity modulating effects of high concentrations of globular proteins (hemoglobin and lysozyme), various Dextrans, and PEGs revealed that these crowding agents differently affected the reaction rates of several decarboxylating enzymes, such as multisubunit enzyme urease, homotetrameric pyruvate decarboxylase, and monomeric glutamate decarboxylase [83]. Here, globular proteins were shown to cause a dramatic increase in the enzymatic activity, whereas a concentration-dependent decrease in the enzymatic activity was observed in the presence of Dextrans and PEGs [83]. Ficoll-70 and Dextran-70 were shown to differently affect the aggregation propensity of a β-rich protein bovine carbonic anhydrase (BCA) [84]. Here, the extent of BCA aggregation was noticeably lower in the presence of Dextran-70 relative to that in the presence of Ficoll-70 (for the solutions containing the same mass/volume percent of these crowders) [84].

Another illustration of the inequality of crowding agents is given by the observation that mixed macromolecular crowding agents affect protein behavior differently than solutions of the individual crowders. For example, although oxidative refolding of the unfolded and reduced hen egg white lysozyme was almost completely abolished at high concentrations of individual crowding agents due to protein aggregation [2], the use of a mixed macromolecular crowding agent containing both bovine serum albumin (BSA) and a polysaccharide resulted in a remarkable increase in the yield of properly folded protein, suggesting that the mixed crowded media could be more favorable for protein folding [85]. Refolding of another protein, rabbit muscle creatine kinase (MM-CK), from its GdmHCl-unfolded state, was differently affected by four single macromolecular crowding agents, Ficoll-70, Dextran-70, PEG-2000, and calf thymus DNA (CT DNA), and three mixed crowding agents containing both CT DNA and a polysaccharide (or PEG-2000), with mixed crowding agents being more favorable for the MM-CK refolding [86].

Finally, a set of inert, flexible, hydrophilic polymers with largely spherical shape (PEG, Dextran and Ficoll) traditionally used for the modeling macromolecular crowding was recently extended to include more rigid polymers with large surface to volume ratio, such as cellulose derivative, hydroxypropyl cellulose (HPC) [87]. The need for this extension was justified by the observation that in addition to spherical biomacromolecules cell contains a number of more rigid (and therefore characterized by the rod-like shapes) biopolymers such as DNA, protein fibers and polysaccharide components of extracellular matrix. Since these rigid polymers have high exposed surface area, they could be more available for interactions with proteins. A systematic study clearly showed that compact, flexible polysaccharides (Dextrans) and more rigid HPCs have rather different effects on structure and aggregation of several proteins with variable degrees of intrinsic disorder and oligomeric state [87]. Based on these studies it has been concluded that different biopolymers influence protein aggregation differently via a complex interplay of several mechanisms including excluded volume effect, solution viscosity, and protein-polymer interaction [87].

Therefore, substantial evidence supports the very important notion that in addition to the excluded volume effects, other factors originating from the presence of high concentrations of “inert” macromolecules in crowded solution should definitely be taken into account. The important players of the crowded environments are direct physical interactions between the crowding agents and proteins, soft interactions, viscosity, perturbed diffusion, and the effects of crowders on solvent properties.

### 4.2. Roles of Direct Interactions of Proteins with Crowding Agents 

This is a rather obvious potential output. If an “inert” bystander molecule is not too inert and possesses at least some affinity to a protein of interest, then direct interactions between such crowding agent and a protein will definitely take place due to the high concentrations of crowder used in the experiments. Obviously, different affinities of a protein to different crowding agents might explain the non-uniform response of such a protein to different crowded environments. For example, the structural stability of human α-lactalbumin (HLA) and transitions of this protein to a molten globule state that can be induced in Ca^2+^-depleted HLA (apo-HLA) at pH 7.0 and 55 °C (*T_m_* of this process in dilute media is 39.7 °C) or at pH 3.0 and 25.0 °C, were differently affected by different crowding agents, such as Ficoll-70, Dextran-70, PEG-2000, and PEG-20000 [88]. The thermal stability of apo-HLA was significantly decreased by high concentrations of PEG-2000 and PEG-20000 (the corresponding *T_m_* decreased by 6 and 4 °C, respectively) thereby leading to the formation of a stable molten globular state at lower temperatures. On the contrary, high concentrations of Dextran-70 and Ficoll-70 dramatically enhanced the thermal stability of apo-HLA (by 12.6 and 3.8 °C, respectively) [88]. The difference in the action of different crowders on thermal stability of apo-HLA was attributed to the fact that no detectable interaction was observed between apo-HLA and Ficoll-70 or Dextran-70, whereas there was a weak, non-specific interaction between the protein and PEG-2000 [88].

Interaction with crowding agents can affect not only the conformational behavior of a protein of interest, but also its functionality. For example, the Ca^2+^/CaM-regulated formation of a complex between the Ca^2+^/CaM-dependent protein kinase II (CaMKII) and the NMDA-type glutamate receptor (NMDAR) subunit GluN2B is differently affected by different macromolecular crowding agents, with efficiency of interaction being reduced by lysozyme but enhanced by BSA [89]. Similar to BSA, high concentrations of Dextran-10, Dextran-70 and poly(vinylpyrrolidone) with molecular weight of 40 kDa (PVP-40) resulted in the more efficient formation of the CaMKII-GluN2B complex, whereas the addition of 100 mg/mL of non-specific rabbit IgG reduced CaMKII binding to GluN2B [89]. The inhibitory effects of lysozyme and IgG in the presence of Ca^2+^ were attributed to the specific or non-specific binding of the regulatory CaM protein to these protein crowders [89].

The rate of the 4-methylumbelliferyl-β-d-*N*-*N'*-*N''*-triacetylchitotrioside ((NAG)(3)-MUF) hydrolysis by hen egg white lysozyme was noticeably decreased due to the human serum albumin (HSA) addition [90]. Although the reaction followed a Michaelis-Menten mechanism under all the conditions employed, the increase in the HSA concentration resulted in the tenfold decrease of the catalytic rate constant, whereas the Michaelis constant was almost independent of HSA in a whole range of albumin concentration employed in the study [90]. The authors showed that this decrease in the lysozyme catalytic efficiency was not due to the substrate association with albumin or because of the HSA-induced macromolecular crowding. Instead, based on the results of the ultracentrifugation analysis, the existence of an HSA-lysozyme interaction has been established, with the decrease in the catalytic rate constant with the HSA concentration being parallel to the decrease of the free lysozyme concentration, suggesting the existence of at least two enzyme populations, the inactive HSA-bound lysozyme and the active free enzyme [90]. This HSA-induced inhibition was explained based on the analysis of the theoretical HSA-lysozyme complex, where the active site residues Glu35 and Asp52 of lysozyme were oriented toward the HSA surface [90]. 

An interesting case study is given by the analysis of oxidative folding of four disulfide bond-containing model peptides with lengths varying from 13 residues (1.4 kDa) to 58-residues (6.5 kDa) (conotoxins GI, PVIIA, and r11a, and bovine pancreatic trypsin inhibitor) in different crowded environments [91]. Here, the addition of high concentrations of polysaccharides did not affect the folding rates or equilibria for these peptides, whereas folding reactions were dramatically accelerated in the presence of protein-based crowding agents (albumin or ovalbumin), even when the concentrations of these crowders were lower than those predicted to provide the excluded volume effects [91]. Since both protein-based crowding agents used in that study contained cysteines, it has been concluded that they might serve as redox-active crowding agents [91].

Based on the analysis of interactability of the globular folded protein, peptidyl-prolyl isomerase Pin1, in *Xenopus laevis* oocytes and in native-like crowded oocyte extract by in-cell NMR spectroscopy, it has been recently concluded that prior to substrate recognition, active Pin1 is involved in numerous nonspecific weak attractive interactions with intracellular proteins [92]. In agreement with several previous studies, the commonly used synthetic crowding agents Ficoll-70 was shown to fail to mimic the intracellular environment being incapable to simulate biologically important weak attractive interactions [92]. The authors stated that these data are in agreement with the model [93] where, due to molecular crowding inside the cells, the majority of globular folded proteins with surface charge properties close to neutral under physiological conditions might be involved in the formation of macromolecular complexes with other sticky proteins [92].

### 4.3. Soft Interactions between Target Proteins and Crowding Agents 

In addition to the aforementioned direct interactions proteins of interest can be involved in non-specific (or soft) interactions with various crowding agents. The presence of such soft interactions is expected to affect the behavior of target proteins in crowded environments [94]. 

To understand the different contributing interactions, concert action of which modulates the response of proteins to the addition of cosolutes in aqueous solution, Harries and colleagues investigated the effect of addition of different cosolutes on folding of a model 16 amino-acid peptide into a β-hairpin [95,96,97]. The authors compared the structure-stabilizing and folding-inducing effects of naturally occurring stabilizing osmolytes, such as sugar (the dissacharide trehalose) and polyols (sorbitol, glycerol, erythriol, manitol, and xylitol) and polymers affecting target peptide via the macromolecular crowding effects (such as PEG-100, PEG-400, PEG-4000, and Dextran-20 and dextran-40). This analysis revealed that although both classes of solutes possessed similar, size-dependent stabilization effect on the peptide, the underlying molecular mechanisms of this stabilization were rather different [95,96,97]. In fact, osmolytes clearly promoted folding of a peptide through an enthalpic stabilization mechanism [95], whereas the mechanisms of the macromolecular crowding agents were concentration-dependent [95,96,97]. Here, at low concentrations, the effects of crowders were predominantly of the entropic nature, whereas at higher concentrations, a significant enthalpic contribution was detected. These observations suggested that in solutions of macromolecular crowders, there is a fine balance between the entropically-driven excluded volume effects (or “depletion interactions”) and some direct “chemical” interactions between the crowder and target protein that can involve significant enthalpic contributions [95,96,97]. The authors hypothesized that the decomposition of folding free energy into its enthalpic and entropic contributions provides new insight into the mechanisms by which different cosolute families may exhibit their stabilizing or destabilizing actions on a target protein [95,96,97].

The capability of the cytoplasm of *Escherichia coli* to promote folding of a destabilized globular protein was recently analyzed by in-cell NMR [98]. In this work, a mutant form of protein L (ProtL), a 7 kDa globular protein from the mesophile *Streptococcus magnus* with seven lysine residues replaced by glutamic acids was expressed in *E. coli*. Although the wild-type protein was characterized by relatively high conformational stability as indicated by the fact that just 0.1% of its molecules populated the denatured state in dilute buffer at room temperature, 84% of the ProtL mutant populated the unfolded state under the same conditions [98]. This mutation-induced destabilization was attributed to the variant’s decreased hydrophobic surface area, and not to the increase in negative charge and was efficiently overcome by adding of Na^+^ or K^+^ salts in dilute solutions [98]. Mutant ProtL remained mostly unfolded in the cytoplasm even when the salt concentration inside *E. coli* was increased [98]. These observations were interpreted in terms of the presence of some nonspecific interactions between the protein and cytoplasmic components. Therefore, stabilizing excluded-volume effects defined by the crowded conditions can be ameliorated by nonspecific interactions between cytoplasmic components [98].

Similarly, based on the analysis of the temperature dependence of NMR-detected amide proton exchange, the entropic and enthalpic contributions of crowding to the stability of ubiquitin were extracted [99]. This analysis revealed that chemical interactions with crowding agents might represent a major contributor to protein stability, often dominating the contribution from the hardcore repulsions (excluded volume effect). Therefore, both soft chemical interactions and hard-core repulsions originating from the excluded volume must be considered when assessing the effects of crowding on protein structure, dynamics, and stability in cells [99].

The mere presence of high concentrations of crowding agents defines the appearance of the excluded volume effects, which are purely entropic hard-core repulsions. On the other hand, the weak interactions of a protein of interest with crowding agents can be repulsive or attractive. Therefore such weak interactions can either enhance or diminish the excluded volume effects [100]. Using the NMR-detected amide proton exchange and various concentrations of the dialyzed, lyophilized, and resuspended *Escherichia coli* cytoplasm as a model crowding agent, the presence of a noticeable destabilization of chymotrypsin inhibitor 2 (CI2) in these crowded solutions was recently found [100]. The destabilization effect increased with increasing cytosol concentration, suggesting that non-specific favorable interaction of cytoplasm with CI2 was able to overcome the stabilizing hard-core repulsions expected from the excluding volume effects [100]. Furthermore, the effects of the cytosol were noticeably stronger than the destabilizing effects of homogeneous protein crowders, providing strong support to the biological significance of weak, nonspecific interactions [100]. 

In the same line of evidence, based on the analysis of the individual opening free energies of the 56-aa B1 domain of protein G (GB1) in living *Escherichia coli* cells it has been concluded that the NMR-detected H/D exchange can measure equilibrium thermodynamic stability of a protein inside the cell at the level of individual amino acid residues under nonperturbing conditions [101]. Here, the thermodynamic stability of a protein was quantified based on the determination of the relative populations of its native and denatured states which are evaluated at the residue level via the analysis of the efficiency of hydrogen–deuterium exchange by NMR. This analysis showed that in comparison to dilute solution data (pH 7.6 and 37 °C) the cellular environment causes significant stabilization of the protein [101]. On the contrary, GB1 was noticeably destabilized in the presence of homogeneous protein crowders (such as lysozyme) *in vitro* due to the prevalence of weak, attractive interactions between anionic GB1 and positively charged lysozyme (pI = 11.3) [101]. The authors also stated that these attractive interactions between the crowding agents (crowders) and the crowded molecules (crowdees) are expected to be destabilizing because, being highly extended, the unfolded protein molecule possesses more reactive surface than the folded one, thereby lowering the free energy of the unfolded state ensemble relative to the folded state [101]. Also, the fact that the cellular environment and concentrated solutions of crowding agents possessed opposite effects on the GB1 stability reemphasized the challenge of recreating the cellular interior by simple means [101]. Based on these observations, the use of the in-cell NMR and the NMR-detected hydrogen-deuterium exchange of quenched cell lysates was proposed as a method for quantification of the stability of globular proteins inside the cells and for the identifications of the residues, which are the most important for the intracellular folding of proteins of interest [101]. 

The effects of weak, nonspecific, chemical interactions between the crowders and protein of interest on structure and stability of a globular protein are expected to depend on the nature of these soft interactions [102]. The effect of steric repulsion on the equilibrium thermodynamic stability of a test protein is expected to be enhanced by repulsive soft interactions, such as interactions between similarly charged species. On the other hand, the conformational stability of a protein is expected to be decreased if attractive soft interactions take place between the crowding molecules and the protein of interest [102]. The ability of anionic proteins from *Escherichia coli* used as crowding agents to affect the conformational stability of the anionic test protein CI2 at pH 7.0 was analyzed to test these hypotheses [102]. Surprisingly, although the bacterial anionic proteins used as crowding agents and the test protein possessed the net charges of the same sign, CI2 was destabilized in anionic crowded environment, suggesting that in addition to the expected charge-charge repulsion there were some weak, nonspecific, attractive interactions between the CI2 and crowding proteins. Therefore, such soft attractive interactions can counterbalance the stabilizing influence of excluded volume effects and overcome the stabilizing charge-charge repulsion [102].

Finally, using a functionalized PEG whose end-group were labeled by Atto488 and Atto565 to measure the mean end-to-end distance by FRET, Gnutt *et al.* recently showed that the model crowding conditions *in vitro* (high concentrations of Ficoll-70 or unlabeled PEG-10000) causes noticeable compaction of this crowding sensor, whereas no contraction was detected in the cell lysates or inside living HeLa cells where the crowding sensor was injected [103]. When this sensor was injected directly into the nucleus, it was significantly more expanded than in the cytosol or diluted buffer, suggesting the existence of even stronger interactions between the crowding sensor and the environment. The authors concluded that weak, transient, and nonspecific interactions (e.g., van der Waals, electrostatic, or hydrophobic forces) between the crowdee and the crowder are efficiently counteracting the excluded-volume effects of the highly crowded cellular milieu [103]. Since this work analyzed the behavior PEG and not a protein in naturally and artificially crowded environments, one can conclude that the made observations are of global nature. 

Observations in this section together with numerous other finding emphasizing the non-equivalency of crowding agents and superiority of natural components over synthetic crowders provide a strong support to a recently proposed hypothesis that crowded environments can be grouped into two different classes, uniform crowding and structured crowding [104]. In this consideration, random crowding conditions created by synthetic particles with a narrow size distribution are classified as uniform crowding, whereas the highly coordinated cellular environments with characteristic clustering and organization of proteins and other macromolecules constitute structured crowding [104]. Some very important features of the structured crowding are the fact that crowders are not necessarily inert, and the presence of a specific organization of such crowded media that might limits the search for appropriate binding partners [104].

### 4.4. Effects of Solvent Viscosity 

Solvent viscosity affects protein diffusion and, in crowded environments, depends on the chemical nature, concentration, and molecular mass of a crowder. Above a certain concentration of crowding agent, the increased solvent viscosity might significantly alter the diffusion rates of a protein of interest. It has been pointed out that a high-mass macromolecular crowding agents not only change the dynamic (or bulk) viscosity of the solution, but could also modulate the microviscosity of the protein environment [105]. Such changes in solvent viscosity might have noticeable effects on the behavior of a protein in crowded environments. For example, based on the analysis of aggregation and fibrillation behavior of several proteins in crowded media with increasing concentration of different crowding agents it has been concluded that above a certain concentration of a crowder, the resulting increase in viscosity defines the decreased diffusion rate of amyloid species leading to the dramatic reduction of the overall aggregation rates [87,106,107]. Since solutions of rigid polymers (such as HPCs) have much higher viscosity they may have stronger effects on the behavior of proteins than solutions of spherical, less rigid polysaccharides (such as Dextrans). In agreement with this hypothesis, protein diffusion was strongly reduced and protein folding and aggregation were noticeably slowed down in the solutions containing HPCs [87].

The presence of noticeable effect of the increased solvent viscosity in crowded environments on protein folding rates has been established in a comprehensive study of the effect of Ficoll on the temperature- and pressure-dependent stability diagram and folding reaction of the globular protein Staphylococcal Nuclease (SNase) [105]. Using FTIR, small-angle X-ray scattering, and calorimetric measurements it has been shown that Ficoll induced noticeable stabilization of SNase, and temperature- and pressure-induced equilibrium unfolding transitions of this protein were markedly shifted to higher temperatures and pressures in solutions containing 30 wt% Ficoll [105]. In the kinetic analysis of SNase refolding, the presence of Ficoll resulted in the dramatic decrease in the SNase folding rate [105]. This was opposite to the expected effect of macromolecular crowding, since folding results in a compaction of the polypeptide chain and the presence of macromolecules near a protein was predicted to accelerate folding reaction through the excluded volume effect. In fact, it was rightly pointed out that when a protein undergoes a folding transition, the transition state or any partially folded intermediate state is more compact than the unfolded state. Therefore, steric repulsion and excluded volume interactions induced by the macromolecular crowding agent should stabilize the folding transition state or partially folded intermediate state, thereby increasing the protein folding rate [105]. Based on these considerations it has been concluded that the increased folding rate in crowded milieu represents a strong evidence in favor of the idea crowding effects on the kinetics of protein folding are dominated by excluded-volume interactions [105]. On the other hand, folding kinetics might also experience the influence of various dynamic effects, such as the modulation of the frictional drag encountered by conformational motions along the folding coordinates. Hence, crowding may also produce a reduction in translational (and, to a less extent, rotational) diffusion constants [105]. Since the decrease in the SNase folding rate was of the order of magnitude of the crowding-induced increase of the solvent microviscosity, it has been concluded that the major effect of the macromolecular crowding agent on the folding kinetics should be based on solvent-induced dynamics changes [105].

Some important conclusions were made based on the analysis of the effects of macromolecular crowding and solvent viscosity on the apparent translational diffusion coefficients (ADCs) of small metabolites with radius of gyration *R_G_* ranging from 0.7 Å to 4.5 Å in solutions with increasing bulk viscosity and different degrees of molecular crowding [108]. The need for this analysis follows from a simple consideration that behavior of a small molecule can be influenced by both bulk solvent viscosity and by the macromolecular crowding that creates obstacles affecting diffusion of said molecule. The authors showed that in agreement with the modified Stokes-Einstein relationship, ADC = *k/R_G_* (1 + 2.5Φ), where *k* is a constant for a given temperature and Φ is an obstruction factor reporting the fractional volume of solution occupied by cosolutes, a measure of the molecular crowding in the solution, for solutions having the same viscosity, metabolite ADCs decreased with increasing concentration of macromolecular crowding agents [108]. 

Another important notion is that the molecular mechanisms of influence of the crowding agents on solvent viscosity are expected to depend on the size of crowding agents. Here, small crowders will affect microviscosity of a solvent, which is the measure of the friction experienced by a single particle undergoing diffusion because of its interaction with its environment at the micrometer length scale. On the other hand, large crowders are expected to affect the macroviscosity of solution which could be considered as a measure of steric hindrance to the diffusion of a protein of interest due to the presence of macromolecular obstacles in solution. The validity of these considerations is supported by the results of a detailed analysis of the formation of a complex between the β-lactamase (TEM) and β-lactamase inhibitor protein (BLIP) in crowded solution using both low and high molecular mass crowding agents such as ethylene glycol (EG), PEG-200, PEG-1000, PEG-3350, PEG-8000, and Ficoll-70 [109]. This analysis revealed that all crowded environments had almost no effect on the dissociation rate constant (*k*_off_) whereas the protein–protein association rate constant (*k*_on_) was noticeably decreased in all crowded solutions. Importantly, the effects of small and large crowders on protein association were fundamentally different [109]. In fact, in solutions containing low mass crowding agents an inverse dependence of *k*_on_ on the solution viscosity was observed, whereas solutions with high mass polymers affected *k*_on_ only slightly, even at viscosities 12-fold higher than water [109]. These observations were interpreted in terms of the formation of a structurally heterogeneous or non-uniform media in the presence of large crowders, where in addition to the volumes occupied by crowding agent, some areas in the solution contain bulk water, and in these areas proteins can diffuse and associate almost as if they were in diluted environments [109]. Very similar results were also reported for the effect of micro- and macromolecular crowding on interaction between cytochrome *f* and plastocyanin from the cyanobacterium *Phormidium laminosum* [110]. Here, high molecular mass viscogens (Ficoll-70 and Dextran-70), had little effect on protein association up to a relative viscosity of 4, whereas low molecular mass viscogens (ethanediol, glycerol, and sucrose) were shown to strongly decrease the bimolecular association rate constant over a similar viscosity range [110]. 

### 4.5. Perturbed Diffusion of Target Proteins in Crowded Milieu

Many dynamic biological processes are governed by the diffusion, which is therefore considered the main transport process inside the cell [111]. Although diffusion is known to result in the dispersion of individual molecules, diffusional motion also mediates reactant encounters therefore serving as the major driving force determining biochemical reactions and pattern formation [112]. Since to a protein of interest diffusing in a crowded environment all the bystander molecules act as obstacles affecting its free diffusion, the *in vivo* diffusion coefficients for globular proteins in living cells are expected and were shown to be strongly decreased compared to the diffusion coefficients of the same proteins in dilute solutions *in vitro* [111,113,114,115,116]. The existence of a complex dependence of the slowing down of protein diffusion on the combination of a protein of interest and crowding agent has been noted [117,118,119,120], and, in several cases, subdiffusive behavior was induced by macromolecular crowding [119,121]. Such subdiffusive motion is manifested by the deviation from the linearity of the mean square displacement of a protein, *v*(*t*), grows in time, which rather showed a power law dependence *v*(*t*) ~ *t*^α^ with α < 1, and this anomaly depends on the size and conformation of the protein of interest and on the total protein concentration of the solution [121]. It has been shown by computer simulations that contrary to the naive expectation that the subdiffusive behavior might compromise efficiency of the diffusive transport and the associated sampling processes in the cell, the probability of finding a nearby target was in fact increased due to the subdiffusion, suggesting that subdiffusion might represent a slower but more reliable diffusive search process inside the cell [122]. 

There is no doubt now that proteins diffuse in crowded solutions anomalously. For example, based on the NMR analysis of the rotational and translational diffusion of a small globular protein, chymotrypsin inhibitor 2 (CI2), in solutions of glycerol, synthetic polymers, proteins, and cell lysates, it has been concluded that there was a noticeable difference in the diffusion behavior of CI2 in solutions of glycerol, synthetic polymers and proteins/cell lysates [120]. In fact, although both translational diffusion and rotational diffusion decrease with increasing viscosity of glycerol solutions and followed respectively the Stokes-Einstein law, *D*_t_ = κ*T/6*πη*r*, and the Stokes-Einstein-Debye law, *D*_r_ = κ*T/8*πη*r*^3^ (where *D*_t_ is the translational diffusion coefficient, *D*_r_ is the rotational diffusion coefficient, η is the solution viscosity, κ is the Boltzmann constant, and *r* is the radius of protein being studied), some significant deviations from the expected behavior were induced by the macromolecular crowding [120]. In fact, synthetic polymers were shown to affect translation more than rotation, causing negative deviation from the Stokes laws [120]. On the other hand, protein crowders and cell lysates had the opposite effect, since they reduced rotational diffusion more than translational diffusion, causing positive deviation from the Stokes laws [120]. The generality of this phenomenon was supported by the observation that the mentioned rotational attenuation was independent of the size and total charge of the protein used as a crowding agent. Based on the NMR relaxation experiments it has been concluded that the presence of weak interactions between the proteins and CI2 represents the major source of the observed difference in the diffusion behavior of CI2 in solutions crowded with synthetic polymers and proteins, suggesting that weak, nonspecific interactions between proteins might fundamentally impact protein diffusion in cells and that synthetic polymers may not be suitable mimics of the intracellular environment [120]. 

Based on the detailed analysis of the diffusion behavior of several proteins of interest in solutions containing high concentrations (up to 200 g/liter) of various bystander or background proteins, it has been concluded that the fractional reduction of the diffusion coefficient of a protein of interest in the presence of a given weight/volume concentration of crowders typically increased with increasing size of protein of interest and with decreasing size of bystander proteins [117]. These observations suggested the diffusive transport of larger proteins and aggregates inside the cells may be slower than in dilute solution by several orders of magnitude [117]. In agreement with this hypothesis, the diffusional mobility of a tracer particle in the cytoplasm was shown to strongly decrease with an increasing radius of the tracked particle, leaving particles with a radius >25–30 nm immobile [114,123,124].

Using quasielastic neutron backscattering, Roosen-Runge *et al.* investigated the protein self-diffusion in crowded aqueous solutions of BSA employing BSA itself as a crowding agent [111]. This analysis revealed that crowding has a very substantial effect on the protein self-diffusion already at the nanosecond time scale and that the diffusion coefficient at biological volume fractions is strongly decreased compared to the dilute limit. These data clearly indicated that in crowded environments, protein dynamics in general and protein diffusion in particular cannot be described solely in terms of excluded volume and confined motions, and hydrodynamic interactions should be taken into account [111]. In agreement with these conclusions, the analysis of binding of a β-lactamase to its protein inhibitor in living HeLa cells revealed that the association rate constants for the wild-type and an electrostatically optimized mutant were only 25% and 50% lower than the corresponding *in vitro* values and no changes in the rate constant were observed for a slower binding mutant [125]. The authors of this study emphasized that changes they detected were much smaller than those anticipated for the highly crowded environment within the cell [125]. 

Comparison of the peculiarities of the translational diffusion of an intrinsically disordered protein α-synuclein (14 kDa) and a well-folded globular protein, chymotrypsin inhibitor 2 (CI2, 7.4 kDa), showed that these proteins behaved rather differently in dilute solution and under crowded conditions (*i.e.*, in the presence of high concentrations (300 g/L) of the synthetic polymers PVP-40 and Ficoll-70 and the globular proteins lysozyme (15 kDa), and BSA (67 kDa) [126]. This analysis revealed that CI2 diffused faster than the disordered protein in dilute solution, whereas under crowded conditions α-synuclein diffused faster than CI2, even though the hydrodynamic volume of α-synuclein is known to be significantly larger than that of CI2 [126]. The authors concluded that one of the key parameters determining protein diffusion under crowded conditions could be the protein shape [126].

### 4.6. Changes in Protein Hydration and Hydration Dynamics

The hydration shell formed by water molecules in the close vicinity of a protein molecule is crucial for protein structure and folding and defines the conformational changes, substrate binding, and molecular recognition [127]. Changes in the protein hydration are known to be related to changes in protein catalytic activity [128,129,130,131,132] and might have a profound effect on protein conformational stability and dynamics [128,132]. Decreased hydration is associated with more rigid structure and altered functionality [128,132]. In an extreme case of dehydration, when a solid protein is dispersed in anhydrous organic solvent, a dramatic increase in thermal stability and striking change of functionality might take place [127]. In fact, in such unnatural media enzyme were shown to acquire remarkable properties such as greatly enhanced stability, radically altered substrate and enantiomeric specificities, molecular memory, and the ability to catalyse unusual reactions [133,134]. 

For example, lipases (whose natural lipolytic reaction in aqueous media is hydrolysis of carboxylic esters), being dispersed in nearly anhydrous organic solvents, were shown to act as efficient catalysts promoting the transesterification reaction between tributyrin and heptanol [135]. Furthermore, these anhydrous enzymes dispersed in the 99.98% organic medium acquired remarkable new properties, becoming extremely thermostable and more selective [135]. Similarly, well-known proteases, such as bovine pancreatic α-chymotrypsin and *Bacillus subtilis* protease (subtilisin Carlsberg), dispersed as solids in anhydrous organic solvents, vigorously acted as catalysts, efficiently conducting the enzymatic transesterification reactions [136]. The rate enhancements of the transesterification reaction achieved by chymotrypsin and subtilisin in octane were of the order of 100 billion-fold [136]. Subsequent analysis of the dependence of catalytic activity of three unrelated enzymes (yeast alcohol oxidase, mushroom polyphenol oxidase, and horse liver alcohol dehydrogenase) in a number of organic solvents as a function of the water content revealed that although enzymes remained catalytically active in a variety of organic solvents, the enzymatic activity was shown to greatly increase in response to an increase in the water content in the solvents (which always remained below the solubility limit) [137]. It has been concluded that organic solvents affect an enzyme primarily due to interactions with the enzyme-bound layer of water rather than with the enzyme itself [137]. 

PEGs are assumed to be preferentially excluded from the hydration shells around proteins or other biomolecules. This exclusion creates osmotic stress, which draws water away from the protein surface or out of a polymer-inaccessible crevice [127]. Osmotic stress has been used to measure changes in the number of water molecules associated with single macromolecules undergoing conformational changes [138]. The catalytic activity of such osmotically stressed enzyme is expected be altered due to the changes in the number of bound water molecules in enzyme, substrate, and in the enzyme-substrate complex (change in *K*_M_, the Michaelis constant) or between the ground state and transition state of the complex (change in *V*_max_, the maximal reaction rate) [127]. In agreement with this hypothesis, addition of PEGs of various molecular weights to solutions of yeast hexokinase increased the affinity of this enzyme for its substrate glucose [138]. These PEG-induced changes in the hexokinase activity were attributed to the indirect action of PEG on an enzyme through PEG-modulated changes in water activity [138]. In fact, evaluation of *ΔN_w_*, the difference in the number of water molecules between the glucose-bound and glucose-free conformations of hexokinase that can be viewed as a release of water from around the protein when HK changes conformation in the direction of cleft closure, revealed that *ΔN_w_* measured at the variety of conditions is dramatically affected by PEGs [138]. *ΔN_w_* was shown to increase from 50 to about 326 water molecules per hexokinase molecule with increasing PEG molecular weight from 300 to 1000 Da and remained constant for PEGs over a wide range of higher molecular weights [138]. Furthermore, an increase in PEG-1000 concentrations resulted in the dramatic decrease in the *ΔN_w_* value from 326 to 50 suggesting that high concentrations of PEG can significantly dehydrate the unbound form of hexokinase [138].

A comparative analysis of the effects of PEG-400 or high concentrations of lysozyme on protein and hydration dynamics of hen egg white lysozyme labeled with a metal-carbonyl vibrational probe covalently attached to a solvent accessible His residue by ultrafast two-dimensional infrared (2D-IR) spectroscopy clearly showed that the effects of the chemically inert polymer and the protein crowder with complex electrostatic profile are different [82]. The uniqueness of technique is in its ability to provide an accurate description of the dynamics of the system through the frequency-frequency correlation function (FFCF), a powerful observable unique to 2D-IR that reports on the equilibrium structural fluctuations that modulate the transition frequency of a probe molecule [82]. The resulting FFCFs exhibit rapid initial picosecond decays due to motion of the hydration water, followed by a significant static offset arising from fluctuations that are too slow to be fully sampled within the experimental window and that are attributed to slow protein fluctuations [139]. Application of this technique revealed that in pure D_2_O, the hydration dynamics occur with a 2.7 ps time constant, which is slower than the dynamics of bulk D_2_O by a factor of 2 [82]. In solution contained high PEG-400 concentrations (80% *v*/*v*) the hydration dynamics was slowed down by nearly a factor of 4, and the protein contribution increased by ~75% relative to the corresponding contribution at pure D_2_O. Furthermore, a dynamic transition manifested by a significant, abrupt slowing of the protein-hydration dynamics was observed around 50% D_2_O [82]. Although the dynamics of both protein and hydration were strongly coupled at all solvent compositions, these dynamics were only weakly coupled to the polymer concentration on either side of this transition [82]. Analysis of the FFCFs in solutions containing non-labeled lysozyme as a crowding agent revealed the presence of similar dynamic transition that occurs at a higher water content (~70%) than when PEG-400 was used as a crowding agent. This difference was attributed to the more significant constraining effect of lysozyme on the surrounding water molecules [82]. The evaluation of the inter-protein distance at which the mentioned dynamic transition took place suggested that there was collective hydration of protein molecules over distances of 30–40 Å which was interpreted in terms of the existence of a dynamical influence of the hydration water extending upward of 15–20 Å from each protein surface [82]. In agreement with these findings earlier Terahertz spectroscopy studies revealed that water dynamics in the solvation layer around one protein molecule is distinct from bulk water out to ≈10 Å and that at high protein concentrations protein solvation layers overlap giving rise to the correlated water network motion beyond 20 Å [140]. A very important conclusion from these studies is that at the expected concentrations of macromolecules inside of cells (300 mg/mL), the majority of water within cells is expected to be involved in slow, collective hydration, with only trace amounts of “bulk-like” water present, despite 50%–70% water content by volume [82]. 

### 4.7. Effects of Crowding Agents on Solvent Properties

One of the interesting aspects of the crowded environment is related to the influence of crowding agents on solvent properties. It is known that the dielectric and thermodynamic properties of water in aqueous solutions of polymers, such as Dextran, Ficoll, PEG, and PVP, change significantly relative to those in pure water [141,142]. Solvent polarity of aqueous media in solutions of Dextran, Ficoll, and PEG was also shown to change depending upon the polymer type and concentration [142]. The use of PEGs as osmotic stressing agents to alter water activity and thereby affect protein hydration was already discussed above [127]. 

An alternative approach of evaluating solvent changes in crowded environments is based on the utilization of aqueous two-phase systems (ATPSes). ATPSes are formed in aqueous mixtures of different water-soluble polymers, or in a solution of a single polymer and certain salt, where, under the appropriate conditions, two or more distinct aqueous phases arise with a well-defined interface [143]. For example, when two specific polymers, such as Dextran and Ficoll, are mixed in water above certain concentrations, the mixture separates into two immiscible aqueous layers. There is a clear interfacial boundary separating two distinct aqueous-based phases, each preferentially rich in one of the polymers, with the aqueous solvent in both phases suitable for biological products [142,144,145]. These systems are unique in that each of the phases typically contains well over 80% water on a molal basis, and yet they are immiscible and differ in their solvent properties [142,146,147,148,149,150,151]. Similar phase separations are known to occur also in aqueous solutions of a single polymer in response to temperature change or salt concentration increase. The aforementioned phase separation in solution of macromolecules depends on their concentration. Typical phase separation thresholds in aqueous mixtures of polymers, proteins, and polysaccharides are within a range of several weight percent of each macromolecule [152,153]. 

It was shown previously for the 1:1 mixture of Dextran and polyvinyl alcohol that even in the case when two polymers are completely compatible (*i.e.*, miscible) in dry state (*i.e.*, in the absence of water), such a mixture in water may form an ATPS [142]. This fact confirms the suggestion [49] that phase separation in aqueous mixtures of two polymers originates from different effects of polymers on the water structure resulting in formation of immiscible hydrogen bond networks of the same aqueous nature. 

An important feature of all known ATPSes is their ability to modulate partitioning of various solutes. In fact, each phase of ATPS provides a distinct solvent environment for small organic molecules, proteins, nucleic acids, RNPs, or other solutes. Differences in solute-solvent interactions in the two phases commonly lead to unequal solute distribution. As a result, a new liquid phase may be specifically enriched or depleted in particular solutes. It has been shown previously [142] and confirmed later that partitioning of various solutes from small organic compounds to proteins and nucleic acids in ATPSs generally does not involve their interactions with phase forming polymers [149,150,154,155,156,157,158,159]. Therefore it has been suggested that unequal distribution of soluble compounds in ATPSs is driven by differences between solute-solvent interactions in the coexisting phases [142]. The partition ratio of an ionizable solute in ATPS may be described as [149]:

(1) $$logK_{s}=S_{s}\Delta \pi *+A_{s}\Delta \beta +B_{s}\Delta \alpha +C_{s}c$$

where *K* is the solute partition ratio; Δπ* is the difference between the solvatochromic solvent dipolarity/polarizability of the coexisting phases, Δα is the difference between the solvatochromic solvent hydrogen-bond donor acidity of the phases, Δβ is the difference between the solvatochromic solvent hydrogen-bond acceptor basicity of the phases; c is the difference between the electrostatic properties of the phases; *S*_s_, *A*_s_, *B*_s_ and *C*_s_ are constants (solute-specific coefficients) that describe the susceptibility of the complementary solute-solvent interactions to the changes in the corresponding solvent properties. The solute-specific coefficients are determined from partition ratios for the solute in a set of ATPS with established solvent properties of the phases by multiple linear regression analysis.

Based on the analysis of partitioning of free amino acids in ten different polymer/polymer ATPSes containing 0.15 M NaCl in 0.01 M phosphate buffer, pH 7.4, a new amino acid scale was proposed [160]. This scale was derived from all the partition ratios values for each amino acid in various ATPSes using the multiple linear regression analysis. This scale is the first scale of amino acids based on their interactions with water. It provides a unique physico-chemical description of amino acids representing their dipole-dipole, hydrogen bonding, and electrostatic interactions with the aqueous environment [160]. Furthermore, it was shown that linear combinations of solute-specific coefficients S_s_, A_s_, B_s_ and C_s_ were correlated with various physicochemical, structural, and biological properties of amino acids [160]. It was also shown that partitioning of closely related proteins in ATPSes is different and that protein partitioning in a set of ATPSes of different compositions can be used to quantify differences between 3D structures of closely related proteins [161]. Furthermore, partitioning of proteins in a set of ATPSs of different ionic compositions was shown to be a useful tool for the quantification of the structural differences between α-synuclein, its variants and globular proteins [162]. Recently, to define mechanisms underlying behavior of protein in ATPSes, partitioning of 15 proteins in Dextran-70-PEG-8000 ATPSes in the presence of 0.01 M sodium phosphate buffer, pH 7.4 and various salt additives (NaCl, CsCl, Na_2_SO_4_, NaClO_4_ and NaSCN) was studied [158]. This analysis revealed that the presence and concentration of salt additives affect the protein partition behavior. The observed differences in protein partition behavior in these systems cannot be explained by differences in protein size or by differences in the relative hydrophobicity and electrostatic properties of the phases. Analysis of about 50 different descriptors of the protein structures suggested that the partition behavior of proteins is determined by the peculiarities of their surfaces (e.g., by the number of water-filled cavities and the averaged hydrophobicity of the surface residues) and by the intrinsic flexibility of the protein structure measured in terms of the B-factor (or temperature factor) [158]. Obviously, in the absence of physical interaction of studied proteins with polymers in different phases of ATPSes, the identified protein surface features define the peculiarities of protein-solvent interactions in different phases. Therefore, the inequality of distribution of soluble compounds in ATPSes, being driven by differences between the solute-solvent interactions in the coexisting phases, clearly represent a direct indication that solvent water in different phases has different properties. Since concentrations of polymers undergoing phase separation are in the range of concentrations used to model molecular crowding (20%–50%), all this indicates that water properties are altered differently in different crowded environments.

The solute-solvent interactions for any given solute are governed by the properties of solvent, though the contributions of different solvent properties would depend on the structure and physicochemical features of a solute. According to Cabot and Hunter [163], most quantitative approaches to the study of solvation phenomena have focused on the use of specially designed spectroscopic probes sensitive to changes in their environment [164,165,166]. The most widely used term for solvent classification is polarity. This is a very poorly defined term which, according to the current definition, is the sum of all possible specific and non-specific interactions between the solvent and any potential solute, excluding interactions leading to chemical transformations of the solute [164,167]. The solute-solvent interactions include multiple types of interactions, such as electrostatic, dipole-dipole, dipole-induced dipole, hydrogen bonding and electron pair donor-acceptor interactions. It is especially important that polarity describes the potential behavior of the solvent in a relationship with the solute which is not an absolute property of the pure solvent [167]. There is a large number of different polarity scales based on different probes and spectroscopic techniques (NMR, IR, UV/Visible absorption and emission spectroscopy, *etc.*) [163]. Since all the polarity scales are estimates based on different assumptions, and since different scales provide different estimates for the same solvent, there is no a single measure of solvent polarity [167]. 

Solvent polarity of aqueous media in solutions of several ATPS forming polymers, such as Dextran, Ficoll, and PEG of different molecular weights was measured using the water soluble carboxylate-substituted anionic betaine Reichard’s solvatochromic dye, and thymol blue [168], or a number of different sulphonephthalein dyes, fluorescein and eosin [169]. Solvent polarity of the phases of Dextran-Ficoll and Dextran-PEG ATPS was estimated with the same solvatochromic dyes and found to be different in the coexisting phases [170]. The differences between solvent polarity of aqueous media in the two phases of polymer/polymer ATPSs together with the differences in the hydrophobic and electrostatic properties of the phases discussed above served as a basis for the suggestion [142,171] that polymer/polymer ATPSs may be viewed as similar to organic solvent-water biphasic systems with the distinction of both phases being of the same aqueous nature.

Any single-parameter polarity scale cannot represent the multitude of possible solute-solvent interactions. Therefore, multi-parameter polarity scales were developed based on Linear Solvation Energy Relationship (LSER) including three scales, such as hydrogen bond donor acidity (α) [172], hydrogen bond acceptor basicity (β) [173], and dipolarity/polarizability (π*) [174]. Combination of these three scales describes ability of a given solvent to participate in solute-solvent interactions; *i.e.*, solvent polarity, much better than any single-parameter polarity scale. This LSER model may be described as:

(2) $$(XYZ)={\left(XYZ\right)}_{o}+s\pi *+a\alpha +b\beta$$

where (*XYZ*) is the solute property (solubility, reaction rate, equilibrium constant, the logarithm of a gas/solvent or solvent/solvent partition coefficient, *etc.*) in a given solvent; *(XYZ)_o_* is the same solute property in a reference state, *s*, *a*, and *b* are the solute-dependent coefficients characterizing the respective influence of the π*, α, and β terms on the (*XYZ*) property under study.

To check the hypothesis that the changes in the solvent properties of aqueous media might be induced by the crowding agents, the solvatochromic comparison method was used to determine the solvent dipolarity/polarizability (π*), hydrogen-bond donor acidity (α), and hydrogen-bond acceptor basicity (β) of aqueous solutions of different polymers (Dextran, PEG, Ficoll, UCON (a copolymer of ethylene glycol and propylene glycol), and PVP) with the polymer concentration up to 40% typically used as crowding agents [159]. Polymer-induced changes in these features were found to be polymer type and concentration specific, and, in case of PEGs, molecular mass specific. Importantly, similarly sized polymers PEG and UCON produced very different changes in the solvent properties of water in their solutions. These two polymers were also shown to induce formation of α-synuclein aggregates with very different morphologies [159]. Importantly, the changed solvent properties in solutions of polymers provided quantitative description of the crowding effects on protein refolding and stability reported in the literature [159]. All these findings provide very strong support to the ideas that the crowding agents do induce changes in solvent properties of aqueous media in crowded environment, and that these changes should be taken into account for crowding effect analysis.

## 5. Conclusions

It is clear now that the excluded volume effect is not the only factor affecting the behavior of various biomolecules (including proteins) in a crowded environment. In addition to the exclude volume effects, which can be described as the hard non-specific steric interactions, important players of the crowded environments are viscosity, perturbed diffusion, direct physical interactions between the crowding agents and proteins, so-called “soft” interactions (such as electrostatic, hydrophobic, and van der Waals interactions) between the crowding agent and the protein, and, most importantly, the effects of crowders on solvent properties. It is very likely that the changes in the solvent properties of aqueous media induced by the crowding agents may be the root of the mentioned “soft” interactions between a protein of interest and a crowding agent [159].

It is shown that macromolecular crowding agents change solvent properties of aqueous media in their solutions. The solvent dipolarity/polarizability, hydrogen-bond donor acidity, and hydrogen-bond acceptor basicity of aqueous media evaluated in solutions of crowding agents are crowding agent-specific and dependent on the concentration of crowders. The further support of notion that steric hindrance or impediment that defines the basis of excluded effect is not the only mechanism affecting behavior of proteins in crowded environments is coming from important observations that PEG and UCON of the same size provoke very different changes in the solvent properties of water in their solutions and induce morphologically different α-synuclein aggregate forms. All this clearly show that crowding agent-induced changes in the solvent properties of aqueous media are important contributors to the macromolecular crowding effects. Therefore, “soft” interactions of a biological macromolecule with crowding agents where proposed to be viewed as the interactions between the macromolecule and aqueous media with solvent properties altered under the crowding agent influence. 

One should keep in mind, however, that the complexity of the intracellular environment extends far beyond the macromolecular crowding. In an outstanding recent review, Selenko and colleagues made an important point that understanding of the peculiarities of protein function inside cells depends on understanding of the specific physical properties of the intracellular environment, where multiple factors are at play [175]. Among these factors shaping the cellular behaviors of proteins are the compositions of the prokaryotic cytoplasm and eukaryotic cytoplasm and nucleoplasm, concentrations of ion and metabolite, dielectric properties of medium, presence of numerous membrane surfaces, intracellular viscosity, and, of course, macromolecular crowding and confinement [175].
